# Observation of Zn-photoprotoporphyrin red Autofluorescence in human bronchial cancer using color-fluorescence endoscopy

**DOI:** 10.1186/s12885-017-3277-6

**Published:** 2017-04-26

**Authors:** Yoshinobu Ohsaki, Takaaki Sasaki, Satoshi Endo, Masahiro Kitada, Shunsuke Okumura, Noriko Hirai, Yoshihiro Kazebayashi, Eri Toyoshima, Yasushi Yamamoto, Kaneyoshi Takeyama, Susumu Nakajima, Isao Sakata

**Affiliations:** 1 Respiratory Center, Asahikawa Medical University, 2-1-1-1 Midorigaoka Higashi, Asahikawa, 078-8510 Japan; 2 Moriyama Memorial Hospital, Asahimachi 2-1-31, Asahikawa, 070-0832 Japan; 3 Porphyrin Lab, Okayama, 700-0086 Japan

**Keywords:** Photodynamic diagnosis, Autofluorescence, Endoscopy, Prophyrin, Zn-photoprotoporphyrin

## Abstract

**Background:**

We observed red autofluorescence emanating from bronchial cancer lesions using a sensitive color-fluorescence endoscopy system. We investigated to clarify the origin of the red autofluorescence.

**Methods:**

The wavelengths of the red autofluorescence emanating from lesions were measured in eight patients using a spectrum analyzer and compared based on pathologic findings. Red autofluorescence at 617.3, 617.4, 619.0, and 617.1 nm was emitted by normal bronchus, inflamed tissue, tissue exhibiting mild dysplasia, and malignant lesions, respectively.

Protoporphyrin, uroporphyrin, and coproporphyrin, the major porphyrin derivatives in human blood, were purchased to determine which porphyrin derivative is the source of red fluorescence when acquired de novo. We synthesized photoporphyrin, Zn-protoporphyrin and Zn-photoprotoporphyrin from protoporphyrin.

**Results:**

Coproporphyrin and uroporphyrin emitted only weak fluorescence. Fluorescence was emitted by our synthesized Zn-photoprotoporphyrin at 625.5 nm and by photoprotoporphyrin at 664.0 nm.

**Conclusions:**

From these results, we conclude that Zn-photoprotoporphyrin was the source of the red autofluorescence observed in bronchial lesions. Zn-protoporphyrin is converted to Zn-photoprotoporphyrin by radiation with excitation light. Our results suggest that red autofluorescence emanating from Zn-photoprotoporphyrin in human tissues could interfere with photodynamic diagnosis using porphyrin derivatives such as Photofrin® and Lazerphyrin® with a sensitive endoscopy system, because color cameras cannot differentiate Zn-photoprotoporphyrin red fluorescence from that of other porphyrin derivatives.

## Background

Components of the human body such as collagen, nicotinamide-adenine dinucleotide phosphate (NADP), and flavin-adenine dinucleotide (FAD), emit fluorescence when irradiated with light of an appropriate excitation wavelength [1, 2]. Normal human bronchial epithelial tissue emits green autofluorescence at a wavelength of ca. 540 nm due to NADP and FAD when excited with 405-nm blue light. This green autofluorescence is less intense in cancer lesions due to thickening of the epithelium, reductions in the levels of the source materials, and absorption of the fluorescence within the lesion. Therefore, cancerous lesions of the bronchus will be demonstrated by a reduction in the intensity of green autofluorescence when the lesions are observed using autofluorescence endoscopy.

Several endoscopy systems have been developed for use in early detection of cancer lesions in the human bronchus. These systems include the LIFE lung [3, 4] (Xillix, Richmond, Canada), SAFE-3000 [5, 6] (Asahi Optical, Tokyo, Japan), D-Light AF [7] (Storz, Tuttlingen, Germany), and AFI (Olympus, Tokyo, Japan). Superior rates of early bronchial carcinoma detection using autofluorescence bronchoscopy (AFB) have been reported in meta-analyses that included data from our study [8, 9]. Although, LIFE lung and SAFE 3000 can detect both red and green fluorescence, only a decrease in the intensity of green autofluorescence in the cancer lesion is detectable using the above-mentioned systems, because their sensitivity is too low to permit visualization of color autofluorescence from human bronchial tissue and because a black and white charged coupled device (CCD) is used in the AFI system [10].

We developed a color fluorescence endoscopy system (PDS-2000 [11, 12]; Hamamatsu Photonics, Hamamatsu, Japan) to observe autofluorescence emanating from human tissues. This system detects both green autofluorescence from normal human organs as well as red autofluorescence from the accumulation of administered porphyrin derivatives. We compared the sensitivity of detection for bronchial cancers and precancerous lesions using this system and found that rate of lesion detection increased significantly, from 54.1 to 89.2%, when AFB was combined with white-light bronchoscopy [13]. During the above clinical study, we detected red autofluorescence emanating from cancer lesions, contact bleeding sites, and blood vessels, and we reported that the red to green autofluorescence ratio (R/G ratio) was significantly higher in the cancer lesions [13].

The accumulation of de novo porphyrin derivatives in cancer tissue, including the accumulation of protoporphyrin IX, has been reported [14, 15]. However, previous reports were based on the results of spectral analyses of resected tumor and drawn blood samples [16–18]. We observed red autofluorescence in human cancer lesions, contact bleeding sites, and the blood vessels of the bronchial wall using a color AFB system. The wavelength of the observed red autofluorescence differed from that reported in previous studies. In the present study, we measured the wavelength of red autofluorescence in order to determine the fluorescent component. This is the first report describing the origin of red autofluorescence observed in human cancer tissues, blood vessels, and contact bleeding sites in living patients using autofluorescence endoscopy.

## Methods

### Autofluorescence endoscopy system

The PDS-2000 fluorescence endoscopy system was developed by Hamamatsu Photonics and Asahikawa Medical University [11–13, 19]. The system includes an intensified color CCD camera, a red-green and blue (RGB) control unit, a source of ca. 405-nm blue light, and a blue-light cut filter. The RGB control unit contains an RGB frame memory, image averaging system, scan converter, and camera control unit. Blue light of an average wavelength of 405 nm generated by a 300-W xenon lamp using a band-pass filter is radiated through the light channel of the fiberscope. The system is connected to an endoscope using an Olympus Endoscopy System attachment.

### Analysis of autofluorescence spectra

Eight Asian patients with high risk of bronchial malignancy were enrolled in the present study. Seven patients had previously treated bronchogenic carcinoma, and one patient had history of bloody sputum (Table 1). Bronchial lesions in eight patients were observed using a bronchofiberscope connected to the PDS-2000 system. Biopsy samples were taken from lesions exhibiting red autofluorescence after measurement of the wavelength emitted from each lesion; samples were also taken from green autofluorescence–emitting tissue of the adjacent normal bronchial wall. The wavelength of lesion autofluorescence was analyzed using a PMA-12 modified color spectrum analyzer (Hamamatsu Photonics). The observation fiber was connected to the PMA-12 and then introduced into the 2-mm channel of the fiberscope. A band-pass filter cutting ca. 405-nm light was used to attenuate blue excitation light from the 300-W xenon lamp. The wavelengths of autofluorescence emanating from the cancer lesions, normal bronchial wall, blood, and blood vessels were determined. This study was approved by the Institutional Review Board of the Asahikawa Medical University (Approval number #237).

**Table 1:** Patients who were enrolled in the present study

Case	Gender	Age	Smoking history/Pack-Year	Diagnosis	Preceding therapy
1	Male	50–59	Current smoker/45	SqCC	Chemo/Ra
2	Male	80–89	Ex-smoker/14	SqCC	PDT
3	Male	70–79	Ex-smoker/36	SqCC	PDT
4	Male	60–69	Current smoker/26	Bloody Sputum	none
5	Male	70–79	Ex-smoker /180	SqCC	PDT
6	Male	70–79	Ex-smoker /105	SqCC	PDT
7	Male	70–79	Current smoker/83	SqCC recurrence	Chemo/Ra, PDT
8	Male	70–79	Current smoker/50	SCLC/SqCC	Chemo/Ra

*SqCC* squamous cell carcinoma, *SCLC* small cell carcinoma, *Chemo* chemotherapy, *Ra* radiation, *PDT* photodynamic therapy

### Synthesis of porphyrin derivatives

**Fig. 1:**
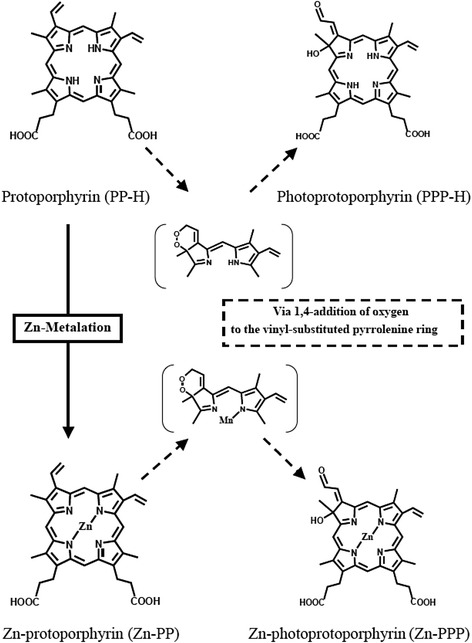
Chemical structures of porphyrin derivatives examined in the present study. Protoporphyrin (PP-H) is converted to photoprotoporphyrin (PPP-H) via 1,4-addition of oxygen to the vinyl substitute. Zn-protoporphyrin (Zn-PP) is converted to Zn-photoprotoporphyrin (Zn-PPP) via 1,4-addition of oxygen to the vinyl substitute. In vitro reported fluorescence wavelengths are 630 nm for PP-H, 664 nm for PPP-H, 585 nm for Zn-PP, and 625 nm for Zn-PPP

Uroporphyrin, coproporphyrin and protoporphyrin were purchased from Wako (Osaki, Japan). Photoprotoporphyrin, Zn-protoporphyrin, and Zn-photoprotoporphyrin were synthesized from protoporphyrin according to previously described methods [20, 21] (Fig. 1).

### Measurement of the wavelengths of fluorescent synthetic porphyrin derivatives

The wavelength of fluorescence emitted by each of our synthesized porphyrins was measured under various conditions and compared with the wavelengths of autofluorescence emanating from the biological specimens.

## Results

### Analysis of the wavelength of autofluorescence emanating from human bronchus

Bright-green autofluorescence was observed in normal human bronchial wall tissue examined using AFB with the PDS-2000 system [13]. Red fluorescing blood vessels were observed in the normal bronchial wall even by AFB. A decrease in the intensity of the green autofluorescence was observed in the bronchial carcinoma lesions.

A total of 29 lesions exhibiting red fluorescence were found in 8 patients. Pathologic diagnosis was normal for 5 lesions and indicated inflammation for 13 lesions, mild dysplasia for 7 lesions, severe dysplasia for 1 lesion, and squamous cell carcinoma for 3 lesions. In the present study, we included lesions exhibiting weak red autofluorescence; therefore, our samples included non-cancerous as well as cancerous lesions. However, it was not difficult to differentiate cancerous from non-cancerous lesions, because the intensity of the red autofluorescence differed. Cancerous lesions were characterized by red autofluorescence by AFB [13].

**Fig. 2:**
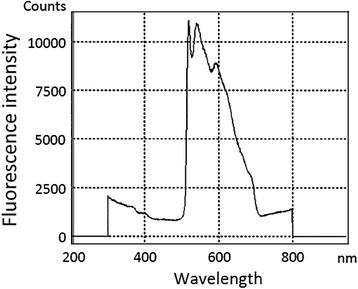
Spectrogram of green autofluorescence observed in the normal bronchial wall with a wavelength of ~540 nm. Data were acquired using a modified PMA-12 (Hamamatsu Photonics, Japan)

**Fig. 3:**
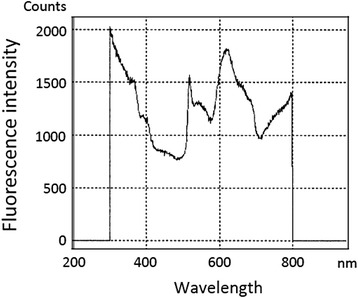
Spectrogram of red autofluorescence observed in squamous cell carcinoma bronchial lesions with a wavelength of ca. 620 nm. Data were acquired using a modified PMA-12 (Hamamatsu Photonics, Japan)

Spectral analyses revealed that the wavelength of the green autofluorescence emanating from the normal bronchial wall tissue adjacent to the 29 lesions was 541.7 ± 0.51 nm (average ± SD, Table 2 and Fig. 2). The average wavelength of the red autofluorescence emanating from the 29 lesions was 617.7 ± 1.31 nm. The intensity of the green autofluorescence was markedly reduced in the squamous cell carcinoma lesions. The cancer lesions appeared red, and spectral analysis of the red autofluorescence showed an average wavelength of 617.1 ± 0.38 nm (Table 2 and Fig. 3). Red autofluorescence associated with bleeding in the bronchial wall resulting from contact with the bronchofiberscope and autofluorescence associated with the blood vessels in the bronchial wall was also observed. The wavelength of red autofluorescence was similar between lesions with different pathologic diagnoses. The wavelengths of green and red autofluorescence according to pathologic diagnosis are listed in Table 2.

**Table 2:** Wavelengths of green and red autofluorescence emanating from bronchial lesions in eight patients (average ± SD)

Pathologic diagnosis	Green autofluorescence (nm)	Red autofluorescence (nm)
Normal (*n* = 5)	541.4 ± 0.00	617.3 ± 0.03
Inflammation (*n* = 13)	541.7 ± 0.49	617.4 ± 0.82
Mild dysplasia (*n* = 7)	542.0 ± 0.67	619.0 ± 2.04
Malignant^a^ (*n* = 4)	541.0 ± 0.00	617.1 ± 0.38

^a^Includes three squamous cell carcinoma and one severe dysplasia

### Analysis of the wavelength of fluorescence emitted by synthetic porphyrin derivatives

To elucidate the source of the red autofluorescence observed by AFB in the bronchial lesions, we tested various porphyrin derivatives found in the human body, which include coproporphyrin, uroporphyrin, and protoporphyrin, and our synthesized photoprotoporphyrin, Zn-protoporphyrin and Zn-photoprotoporphyrin. Coproporphyrin and uroporphyrin emitted only weak fluoresce when excitation light was applied. Protoporphyrin, photoprotoporphyrin, Zn-protoporphyrin, and Zn-photoprotoporphyrin reportedly emit fluorescence at 630, 664, 585, and 625 nm, respectively, when excited with 400-nm light (Fig. 1).

**Fig. 4:**
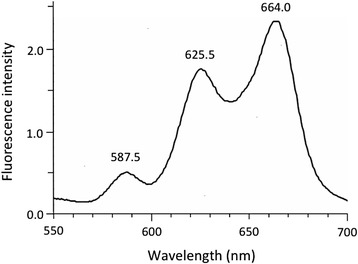
Our synthesized Zn-photoprotoporphyrin and photoprotoporphyrin were dissolved in 5% albumin solution and excited with 400-nm light. Fluorescence emitted by synthetic porphyrin derivatives at wavelengths of 587.5, 625.5, and 664.0 nm. We concluded that the 587.5-nm fluorescence was from albumin, the 625.5-nm fluorescence was from Zn-photoprotoporphyrin, and the 664.0-nm fluorescence was from photoprotoporphyrin

Our synthesized Zn-photoprotoporphyrin and photoprotoporphyrin were dissolved in 5% albumin solution and excited with 400-nm light. Fluorescence at wavelengths of 587.5, 625.5, and 664.0 nm was observed (Fig. 4). We added 5% albumin to the solution, because it was reported that the biochemical/biological environment, which might alter the quantum yield and lifetime of the fluorophore(s) [22]. However, 5% albumin did not seem to alter wavelength of the fluorescence. We concluded that the 587.5-nm fluorescence was from albumin, the 625.5-nm fluorescence was from Zn-photoprotoporphyrin, and the 664.0-nm fluorescence was from photoprotoporphyrin. In the present study, our synthesized Zn-protoporphyrin emitted 578-nm fluorescence (data not shown). These results suggested that Zn-protoporphyrin in living patients is converted to Zn-photoprotoporphyrin upon excitation with 400-nm light, and emits 625.5-nm fluorescence [23]. The difference between the 617.7-nm fluorescence observed in the human bronchus and the 625.5-nm fluorescence observed in the above experiment can be attributed to differences between in vivo and in vitro conditions. Therefore, we concluded that the source material of the red autofluorescence observed in cancer lesions, blood vessels, and contact bleeding sites using the PDS-2000 system was Zn-photoprotoporphyrin. The red fluorescence from Zn-photoprotoporphyrin could be detected visibly using the fluorescence endoscopy system in cancer lesions in which the intensity of green autofluorescence from normal tissue decreased, as well as in blood vessels and contact bleeding sites.

## Discussion

Detection of red autofluorescence in cancers of the bladder, stomach, and lung has been reported. High-performance liquid chromatography (HPLC) analysis of tissues from patients with these cancers revealed substances emitting faint red fluorescence [17, 18]. The source of this red fluorescence has been attributed to the de novo accumulation of porphyrins [16, 24]. However, this hypothesis has not been confirmed. We observed bronchogenic cancer lesions using a color fluorescence endoscopy system and found an increase in the R/G ratio in the cancer lesions [13]. Kluftunger et al. [25] reported increase of R/G ratio greater than 1.5 times the control, fluorescence imaging correctly identified areas of hyperplasia, dysplasia, CIS and invasive cancer using DMBA-induced hamster cheek pouch model. In our previous study, R/G ratio in bronchogenic cancer was significantly greater than those in normal bronchial wall due to decrease of green fluorescence and increase of red fluorescence in the cancer lesions [13]. This red fluorescence was also observed in blood vessels as well as in fresh contact bleeding sites in the bronchial wall. We found that the wavelength of the red fluorescence was 617.7 nm, and the source of the red fluorescence in the present study was identified as Zn-photoprotoporphyrin. Zn-photoprotoporphyrin seems to be formed from Zn-protoporphyrin following irradiation with 405-nm blue light. However, it was difficult to extract porphyrin analogues from small biopsy specimen from the bronchial wall.

De novo protoporphyrin IX has been implicated as a source of the red autofluorescence associated with cancerous tissues. Moesta et al. reported the emission of red fluorescence from colorectal cancers [18]. They analyzed chemical extracts of involved lymph nodes using reversed-phase HPLC and found a substance emitting 630-nm fluorescence. They concluded that protoporphyrin IX was the source of the red autofluorescence in these involved lymph nodes. Croce et al. reported naturally occurring porphyrins in a spontaneous tumor-bearing mouse model [17]. They reported substantial levels of protoporphyrin IX in tumor, spleen, liver, and plasma samples.

Protoporphyrin IX is formed from 5-aminolevulinic acid; however, its concentration in normal human tissues is low [26]. In addition, the wavelength of protoporphyrin IX fluorescence is 635 nm when excited with 405-nm light [27]. These data suggest that the 617.7-nm autofluorescence emanating from cancer lesions, blood, and blood vessels in the present study was from a source other than protoporphyrin IX. The human body must therefore naturally contain a substance that emits strong, red autofluorescence. The present study was conducted to identify the source of the 617.7-nm red autofluorescence observed in previous studies.

The major porphyrin derivatives found in normal human blood are uroporphyrin, coproporphyrin, and Zn-protoporphyrin. Normal blood levels of porphyrins are 0–1.0 μg/dl for total porphyrin, <2 μg/dl for coproporphyrin, 16–60 μg/dl for protoporphyrin, <2 μg/dl for uroporphyrin [28] and 23 μg/dl for Zn-protoporphyrin [29]. Zn-protoporphyrin reportedly emits fluorescence at 585 nm, but our synthesized Zn-protoporphyrin examined emitted 578-nm red fluorescence. This wavelength differed from the 617.7-nm fluorescence observed in bronchial cancer lesions, blood vessels, and contact bleeding sites. We then examined our synthesized Zn-photoprotoporphyrin and photoprotoporphyrin by dissolving them in 5% albumin solution to mimic the conditions of the human body, and fluorescence from both of these porphyrin derivatives was detected. Zn-photoprotoporphyrin and photoprotoporphyrin emitted fluorescence at 625.5 and 664.0 nm, respectively, and we therefore concluded that the red fluorescence emanating from bronchial cancer lesions, blood vessels, and contact bleeding sites in the present study was associated with Zn-photoprotoporphyrin. This conclusion is plausible, as the difference in wavelengths was acceptable, considering the measurement method and the in vivo and in vitro conditions. In the human body, Zn-protoporphyrin (emitting 578-nm red fluorescence) seems to become Zn-photoprotoporphyrin (emitting 625.5-nm red fluorescence) following irradiation with 405-nm excitation light via photooxidation [30]. It is known that cancer lesions emit bi-phasic red fluorescence during photodynamic therapy (PDT) forming protoporphyrin photoproducts [31, 32]. This bi-phasic red fluorescence is emitted by protoporphyrin IX, which emits 636 nm red fluorescence, and photoprotoporphyrin, which emits 674 nm red fluorescence in case of PDT using 5-ALA [32]. In PDT, protoporphyrin becomes photoprotoporphyrin upon laser irradiation. Our present report is the first to describe the origin of red autofluorescence emanating from cancer lesions, blood vessels and fresh contact bleeding sites in living patients.

Autofluorescence endoscopy revealed a decrease in the intensity of the green fluorescence emanating from normal human tissue. Autofluorescence endoscopy typically utilizes AFI and D-light AF systems. However, it is difficult to detect the red autofluorescence that emanates from cancer lesions, blood, and blood vessels using either system. This has led some researchers to conclude that human blood and blood vessels do not emit autofluorescence or emit only weak autofluorescence associated with hemoglobin. However, we found that red autofluorescence could be clearly detected using a sensitive autofluorescence endoscopy system such as the PDS-2000. We have observed red autofluorescence not only in bronchogenic carcinoma but also in tumors metastasized from breast, colon, and pancreatic cancers. We developed a new autofluorescence endoscopy system using an EM-CCD, PDS-TriMode (FLOVEL, Tachikawa, Japan), based on the PDS-2000 technology. The PDS-TriMode is a high-vision system, and its sensitivity is greater than that of the PDS-2000. The PDS-TriMode is capable of clearly detecting not only decreases in green autofluorescence but also abnormal red autofluorescence emanating from cancer lesions, blood, and blood vessels.

Analysis of the wavelength of red fluorescence can provide very important information. When 5-aminolevulinic acid (5-ALA) is orally administered, levels of protoporphyrin IX (which emits 635-nm red fluorescence when excited with 405-nm light) increase in cancer tissues [33, 34]. Photofrin® and Lazerphyrin® have been approved and are currently used in PDT in Japan. In cancer tissues, Photofrin® and Lazerphyrin® emit 640- and 664-nm red fluorescence, respectively. These drugs are also used to detect cancerous tissue in photodynamic diagnosis (PDD). It is obviously difficult to differentiate 617.7-nm red autofluorescence emanating from the blood from 635-, 640-, and 664-nm red fluorescence using a sensitive color CCD camera. Attempts to do so could lead to false results in PDD. Our results indicate that reduction in the intensity of 617.7-nm red autofluorescence emanating from the blood is necessary for reliable PDD using porphyrin derivatives and 5-ALA.

## Conclusions

We conclude that Zn-photoprotoporphyrin was the source of the red autofluorescence observed in bronchial lesions. Zn-protoporphyrin is converted to Zn-photoprotoporphyrin by radiation with excitation light. Our results suggest that red autofluorescence emanating from Zn-photoprotoporphyrin in human tissues could interfere with photodynamic diagnosis using porphyrin derivatives such as Photofrin® and Lazerphyrin® with a sensitive endoscopy system, because color cameras cannot differentiate Zn-photoprotoporphyrin red fluorescence from that of other porphyrin derivatives.

## Abbreviations

5-ALA: 5-aminolevulinic acid
AFB: Autofluorescence bronchoscopy
CCD: Charged coupled device
FAD: Flavin-adenine dinucleotide
HPLC: High-performance liquid chromatography
NADP: Nicotinamide-adenine dinucleotide phosphate
PDT: Photodynamic therapy
R/G ratio: Red to green autofluorescence ratio
RGB: Red, green and blue

## References

[1] Lakowicz JR (1983). Principles of fluorescence spectroscopy.

[2] Schomacker KT, Frisoli JK, Compton CC, Flotte TJ, Richter JM, Nishioka NS (1992). Ultraviolet laser-induced fluorescence of colonic tissue: basic biology and diagnostic potential. Laser Surg Med.

[3] Palcic B, Lam S, Hung J, MacAulay C (1991). Detection and localization of early lung cancer by imaging techniques. Chest.

[4] George PJ (1999). Fluorescence bronchoscopy for the early detection of lung cancer. Thorax.

[5] Kakihana M, Li KK, Okunaka T, Furukawa K, Hirano T, Konaka C (1999). Early detection of bronchial lesions using system of fluorescence endoscopy (SAFE) 1000. Diagn Ther Endosc..

[6] Adachi R, Utsui T, Furusawa K (1999). Developement of the autofluorescence endoscope imaging system. Diagn Ther Endosc..

[7] Leonhard M (1999). New incoherent autofluorescence/fluorescence system for early detection of lung cancer. Diagn Ther Endosc..

[8] Chen W, Gao X, Tian Q, Chen L (2010). A comparison of autofluorescence bronchoscopy and white light bronchoscopy in detection of lung cancer and preneoplastic lesions: a meta-analysis. Lung Cancer.

[9] Sun J, Garfield DH, Lam B, Yan J, Gu A, Shen J (2011). The value of autofluorescence bronchoscopy combined with white light bronchoscopy compared with white light alone in the diagnosis of intraepithelial neoplasia and invasive lung cancer: a meta-analysis. J Thorac Oncol.

[10] Aihara H, Sumiyama K, Saito S, Tajiri H, Ikegami M (2009). Numerical analysis of the autofluorescence intensity of neoplastic and non-neoplastic colorectal lesions by using a novel videoendoscopy system. Gastrointest Endosc.

[11] Ohsaki Y, Nishigaki Y, Takeyama K, Nakanishi K, Ide H, Matsumoto H (2000). Visualization of cancer using high sensitive fluorodynamic camera and fiber-optic endoscope. Porphyrins.

[12] Ohsaki Y, Takeyama K, Nakao S, Tanno S, Toyoshima E, Nakanishi K (2001). Detection of photofrin fluorescence from malignant and premalignant lesions in the bronchus using a full-color endoscopic fluorescence imaging system: a preliminary report. Diagn Ther Endosc.

[13] Nakanishi K, Ohsaki Y, Kurihara M, Nakao S, Fujita Y, Takeyama K (2007). Color auto-fluorescence from cancer lesions: improved detection of central type lung cancer. Lung Cancer.

[14] Ghadially FN, Neish WJP (1960). Porphyrin fluorescence of experimentally produced squamous cell carcinoma. Nature.

[15] Lycette RM, Leslie RB (1965). Fluorescence of malignant tissue. Lancet.

[16] Bottiroli G, Croce AC, Marchesini R, Pignoli E, Tomatis S, Cuzzoni C (1995). Natural fluorescence of normal and neoplastic human colon: a comprehensive 'ex vivo' study. Lasers Surg Med.

[17] Croce AC, Santamaria G, De Simone U, Lucchini F, Freitas I, Bottiroli G (2011). Naturally-occurring porphyrins in a spontaneous-tumour bearing mouse model. Photochem Photobiol Sci.

[18] Moesta KT, Ebert B, Handke T, Nolte D, Nowak C, Haensch WE (2001). Protoporphyrin IX occurs naturally in colorectal cancers and their metastases. Cancer Res.

[19] Shibukawa K, Miyokawa N, Tokusashi Y, Sasaki T, Osanai S, Ohsaki Y (2009). High incidence of chromosomal abnormalities at 1p36 and 9p21 in early-stage central type squamous cell carcinoma and squamous dysplasia of bronchus detected by autofluorescence bronchoscopy. Oncol Rep.

[20] Dolphin D, Sivasothy R (1981). The preparation of porphyrin S-411 (dehydrocoproporphyrin) and harderoporphyrin from protoporphyrin IX. Can J Chem.

[21] Nakae Y, Fukusaki E-I, Kajiyama S-I, Kobayashi A, Nakajima S, Sakata I (2005). Syntheses and screening tests of new chlorin derivatives as photosensitizer. J Photochem Photobiol A.

[22] Ramanujam N (2000). Fluorescence spectroscopy of neoplastic and non-neoplastic tissues. Neoplasia.

[23] Falk JE, Smith KM. Porphyrins and metalloporphyrins. Revised ed. Elsevier Science; 1975. p. 688–689.

[24] Ghadially FN, Neish WJP, Dawkins HC (1963). Mechanisms involved in the production of red fluorescence of human and experimental tumors. J Pathol Bacteriol.

[25] Kluftinger AM, Davis NL, Quenville NF, Lam S, Hung J, Palcic B (1992). Detection of squamous cell cancer and pre-cancerous lesions by imaging of tissue autofluorescence in the hamster cheek pouch model. Surg Oncol.

[26] Sachar M, Anderson KE, Ma X (2016). Protoporphyrin IX: the good, the bad, and the ugly. J Pharmacol Exp Ther.

[27] Nakai Y, Anai S, Onishi S, Masaomi K, Tatsumi Y, Miyake M (2015). Protoporphyrin IX induced by 5-aminolevulinic acid in bladder cancer cells in voided urine can be extracorporeally quantified using a spectrophotometer. Photodiagn Photodyn Ther.

[28] Porphyrins-blood test. MedlinePlus. http://medlineplus.gov/ency/article/003372.htm

[29] Suga RS, Fischinger AJ, Knoch FW (1981). Establishment of normal values in adults for zinc protoporphyrin (ZPP) using hematofluorometer: correlation with normal blood lead values. Am Ind Hyg Assoc J.

[30] Cox G, Whitten DG (1982). Mechanisms for the photooxidation of protoporphyrin IX in solution. J Am Chem Soc.

[31] König K, Schneckenburger H, Rück A, Steiner R (1993). In vivo photoproduct formation during PDT with ALA-induced endogenous porphyrins. J Photochem Photobiol B.

[32] Robinson DJ, de Bruijn HS, van der Veen N, Stringer MR, Brown SB, Star WM (1998). Fluorescence photobleaching of ALA-induced protoporphyrin IX during photodynamic therapy of normal hairless mouse skin: the effect of light dose and irradiance and the resulting biological effect. Photochem Photobiol.

[33] Kitada M, Ohsaki Y, Matsuda Y, Hayashi S, Ishibashi K (2014). Photodynamic diagnosis of malignant pleural diseases using the autofluorescence imaging system. Ann Thorac Cardiovasc Surg.

[34] Kitada M, Ohsaki Y, Matsuda Y, Hayashi S, Ishibashi K (2015). Photodynamic diagnosis of pleural malignant lesions with a combination of 5-aminolevulinic acid and intrinsic fluorescence observation systems. BMC Cancer.

